# The Importance of Intact Senses in Mating and Social Assessments Made by Deaf Individuals

**DOI:** 10.1007/s10508-021-02016-6

**Published:** 2021-10-12

**Authors:** Anna Oleszkiewicz, Paulina Idziak, Marta Rokosz

**Affiliations:** 1 Institute of Psychology, University of Wroclaw, ul. Dawida 1, 50-527 Wrocław, Poland; 2 Taste and Smell Clinic, Technische Universitat Dresden, Dresden, Germany; 3 Faculty of Psychology, SWPS University of Social Sciences and Humanities, Wrocław, Poland

**Keywords:** Mate selection, Attractiveness, Deafness, Sensory modalities

## Abstract

Social perception is a multimodal process involving vision and audition as central input sources for human social cognitive processes. However, it remains unclear how profoundly deaf people assess others in the context of mating and social interaction. The current study explored the relative importance of different sensory modalities (vision, smell, and touch) in assessments of opposite- and same-sex strangers. We focused on potential sensory compensation processes in mate selection (i.e., increased importance of the intact senses in forming impressions of an opposite-sex stranger as a potential partner). A total of 74 deaf individuals and 100 normally hearing controls were included in the study sample. We found diminished importance of vision and smell in deaf participants compared with controls for opposite- and same-sex strangers, and increased importance of touch for the assessment of same-sex strangers. The results suggested that deaf people rely less on visual and olfactory cues in mating and social assessments, highlighting a possible role of sign language in shaping interpersonal tactile experience in non-romantic relationships.

## Introduction

Socially relevant information can be acquired through vision (Fan et al., 2004, 2005), audition (Hughes & Miller, 2016; McAleer et al., 2014), olfaction (Roberts et al., 2005), and touch (Karremans et al. 2010; Kestenbaum & Nelson, 1992; Kupers & Ptito, 2014), indicating that interpersonal perception is multimodal (Groyecka et al., 2017). Visual perception is generally considered to be the most important sensory input because visual signals are easily accessible and can calibrate auditory, olfactory, and tactile cues (Krupp, 2007). However, increasing evidence suggests the significance of auditory cues in social perception. From an evolutionary perspective, sexual dimorphism in voice pitch serves to communicate formidability with lower fundamental frequency (*F*0) (Puts et al., 2019). Research regarding the role of auditory signals in attractiveness perception suggests that females generally prefer deeper male voices (Collins, 2000; Feinberg et al., 2005a, b), while males prefer higher-frequency female voices (Feinberg et al., 2005a, b), and attribute them to younger females (Collins & Missing, 2003). These preferences stem from associating deeper male voices with larger, more masculine bodies, and higher-pitched female voices with smaller, and more feminine bodies (Pisanski et al., 2012).

Recent studies suggest that voice preferences in the context of mating are relatively stable across the ovulatory cycle (Jünger et al., 2018a, b). The human voice prompts socially relevant information, including trustworthiness, competence and warmth, even in the absence of additional visual cues (Oleszkiewicz et al., 2016, 2017; Pisanskiet al., 2016). However, no relationship has been found between lower voice pitch in men and perceived economic trustworthiness, despite such a relationship being observed for mating-related trustworthiness (self-reported relationship infidelity) (Schild et al., 2020). The importance of auditory cues in social perception was highlighted in a recent study of blind individuals, concluding that the loss of vision results in enhanced importance of audition in social assessments, and that hearing is the second-most crucial modality in human interaction (Sorokowska et al., 2018). In a mate-choice situation, such as speed-dating, individuals tend to modulate their voice to appear more attractive (Pisanski et al., 2018). Overall, the aforementioned evidence indicates that audition is important for both self-presentation and building impressions of others. The importance of auditory cues in social perception raises the question of how social perception is constructed under conditions with no auditory input. Because blind people rely on auditory signals in mate selection more heavily than sighted individuals (Sorokowska et al., 2018), we expected to observe an analogous phenomenon in deaf people, despite the importance of intact modalities possibly being different.

The human brain shows remarkable plasticity, enabling compensation for the lack of auditory input. Deaf individuals show stronger event-related potential responses to visual and non-painful tactile stimulation compared with normally hearing controls (Güdücü et al., 2019). A compensatory effect among deaf individuals in cognitive tasks has been found in terms of visual abilities: non-hearing signers exhibit an enhanced ability to detect subtle changes in facial expression (McCullough & Emmorey, 1997), better visual attention (Sladen et al., 2005) including shorter reaction times to visual stimuli (Bottari et al., 2010; Heimler & Pavani, 2014; Hong Lore & Song, 1991; Reynolds, 1993), better perception in peripheral vision (Proksch & Bavelier, 2002) and greater attention to motion (Dye & Bavelier, 2013). However, Voss et al. (2010) conducted a review reporting that the abilities of non-hearing subjects with regard to visual contrast sensitivity, motion velocity, and motion sensitivity were comparable to those of normally hearing people. Previous studies have reported comparable olfactory acuity (Sorokowska et al., 2020) and diminished olfactory performance (Diekmann et al., 1994; Güdücü et al., 2016) as a consequence of hearing loss. Deaf people have been reported to exhibit a similar level of disgust elicited by the body odors of others (Stefańczyk & Oleszkiewicz, 2020) compared with hearing controls. Processing tactile stimuli has been found to activate the primary hearing cortex (Auer et al., 2007; Levänen et al., 1998) and somatosensory cortex (Güdücü et al. 2019) in deaf individuals. The superiority of deaf individuals over hearing controls has been revealed in complex tasks such as identifying emotions expressed with vibrotactile stimuli (Sharp et al., 2020) followed by stronger neural responses to a tactile stimulus (Güdücü et al. 2019).

To date, the role of intact senses in shaping mating preferences in deaf individuals remains unexplored. Based on evidence regarding the superiority of deaf individuals in socially relevant tasks involving the processing of visual and vibrotactile stimuli, such as detection of subtle changes in facial expression (McCullough & Emmorey, 1997), visual attention (Sladen et al., 2005), shorter reaction times to visual stimuli (Bottari et al., 2010; Heimler & Pavani, 2014; Hong Lore & Song, 1991; Reynolds, 1993), and identification of emotion from vibrotactile stimulation with music (Sharp et al., 2020), we expected to observe increased importance of visual and tactile stimuli in assessing the attractiveness of other people as a consequence of increased visual, and tactile performance. On the contrary, based on previous evidence indicating a lack of sensory compensation in the chemosensory domain, we did not expect to observe increased importance attributed by deaf individuals to olfactory cues in the mating context. To address these hypotheses, we investigated the importance of visual, olfactory, and tactile cues in the perception of attractiveness of an opposite-sex stranger (potential partner) and a same-sex stranger, in a large sample of profoundly deaf subjects.

## Method

### Participants

We determined the required sample size using G*Power software (Faul et al., 2007). For a repeated measures analysis of variance (described in detail in the Statistical analyses section) with four groups (deafness * sex), to obtain statistical power of 0.80 with an alpha level of 0.05 to detect medium-to-large effects of *f*^2^ = 0.25 (Sorokowska et al., 2018), the projected sample size was at least 179 participants. The current study was carried out with a sample of 174 Polish natives. Seventy-four deaf individuals aged between 16 and 55 years (*M*_age_ = 30.74 ± 11.46; 37 females) were diagnosed with profound deafness, meaning that their hearing threshold for both ears exceeded 90 dB (the noise level of a landing Boeing 737 aircraft or working newspaper press), translating to practically nonexistent hearing ability in social interactions. Among the participants, 31 had no hearing aid, 10 used cochlear implants, and 32 used hearing aids. Sixty-two participants were categorized as early-deaf (before 6 years of age, which is recognized as the age threshold for language acquisition) and four were categorized as late-deaf (auditory loss onset after 6 years of age). The onset of auditory loss was independent from the type of hearing aid, *χ*^2^(2) = 5.19, *p* = 0.08. Among the participants, 57 were raised by two hearing parents, 13 were raised by two deaf parents and one was raised by one deaf parent. To further confirm profound deafness in a social context, we performed auditory testing comprising vocal audiometry and the triplet test. Participants performed the test while wearing a hearing aid. For this purpose, we used web-based software (e-audiologia.pl) (Masalski & Kręcicki, 2013; Masalski et al., 2014) and Sennheiser HD 280 Professional headphones. Vocal audiometry is designed to test participants’ ability to understand common and simple words. The participant hears a word in the headphones and is required to identify it among five distractors (i.e., words with different meanings) displayed on the computer screen. In the triplet test, participants were required to repeat a span of three digits presented in headphones under conditions of varying sound-to-noise ratio. After hearing the stimulus, participants were asked to repeat the digit span using digital keyboard displayed on the computer screen. Seventy-four profoundly deaf participants whom we examined did not exceed a performance level of 50% for understanding words in vocal audiometry in either ear, regardless of the decibel hearing level (dbHL) and only two reached 50% of understanding in the triplet test at a sound-to-noise ratio of − 6 and − 10.5 points. The control sample consisted of 100 normally hearing people aged between 16 and 57 years (*M*_age_ = 31.13 ± 11.59; 53 females), who were matched in terms of age, *t*(172) = 0.22, *p* = 0.83 and sex, *χ*^2^(1) = 0.15, *p* = 0.70 to eliminate potential confounds resulting from variance in sensory performance and the meaning attributed to the signals acquired by the intact senses by men and women: vision (Bimler et al., 2004; Gittings & Fozard, 1986; Pitts, 1982), olfaction (Brand & Millot, 2001; Doty & Cameron, 2009; Oleszkiewicz et al., 2019; Ottaviano et al., 2016; Sorokowska et al., 2014, 2019) and touch (Dinse et al., 2006; Falling & Mani, 2016; Nguyen et al., 1975; Russo et al., 2020; Wickremaratchi & Lleweln, 2006). Hearing ability in the control group was qualified by reaching 100% intelligibility in vocal audiometry and the triplet test. Participants received a modest financial remuneration.

### Procedure and Measures

Data were collected with pen and paper interview during individual sessions in a dedicated laboratory. Deaf participants who could not read instructions or the experimenter’s lips were accompanied by a professional sign language translator. At the beginning of the session, an experimenter presented each participant with a consent form and briefly explained the protocol. Participants were instructed that they would be asked a series of questions relating to the importance of different modalities in assessments of strangers. To examine differences in social sensory assessment between deaf and hearing individuals, the same questions were asked about an opposite-sex stranger (potential partners) and a same-sex stranger. The experimenter explained that the term potential partner in this context referred to a mate (a romantic or sexual partner). The survey was based on the Sensory Stimuli and Sexuality Survey (Herz & Cahill, 1997). The survey items were: When meeting an opposite-sex stranger (a potential partner)/a same-sex stranger, what draws my attention is: (1) The way they look, (2) The way they feel (regarding skin touch), (3) The way they smell. Participants were asked to rate each modality using a 5-point Likert scale, with 1 indicating “definitely not” and 5 indicating “definitely yes”. Furthermore, the participants were asked an alternative forced choice question to indicate Which of these modalities do you consider the most important in the overall assessment? for which there were three possible answers: Appearance, Touch or Smell. An additional alternative forced choice question was asked regarding an opposite-sex stranger: Which of these modalities do you consider the most important when assessing the attractiveness of this person? with three possible answers: Appearance, Touch or Smell. The order of questions referring to the opposite-sex stranger (potential partner) and the same-sex stranger was random.

### Statistical Analyses

Statistical analyses were performed using SPSS v. 25 with *p* < 0.05 set as the level of significance. Initially, we tested whether (1) hearing support or (2) presence of the sign language translator influenced the responses. We ran two mixed factor design models with (1) modality (vision vs touch vs smell) and target (opposite-sex stranger vs same-sex stranger) as within-subjects factors and hearing support (hearing aid vs cochlear implant vs none) as a between-subjects factor; (2) modality (vision vs touch vs smell) and target (opposite-sex stranger vs same-sex stranger) as within-subjects factors and the presence of sign language translator (yes vs no) as a between-subjects factor. Further, we performed an omnibus analysis of variance with a mixed factor design. Within the tested model we included modality (vision vs touch vs smell), and target (opposite-sex stranger vs same-sex stranger) as within-subjects factors and participants’ sex (male vs female), and deafness (deaf vs hearing) as a between-subjects factor. For multiple comparisons, we applied Bonferroni corrections. Consequently, based on the observed effects, we performed analogous models for each target separately. Furthermore, we examined independence of the most important modality in overall/attractiveness assessment from deafness using chi-square tests.

## Results

Neither the use of hearing support, *F*(2, 70) = 1.21, *p* = 0.31, *ŋ*^2^ = 0.03, nor the presence of a sign language translator, *F*(1, 71) = 1.62, *p* = 0.21, *ŋ*^2^ = 0.02 had an effect on responses. In the course of an omnibus mixed factor analysis of variance, we observed a main effect of a target, *F*(1, 170) = 43.73, *p* < 0.001, *ŋ*^2^ = 0.21, wherein overall assessments of the importance of modalities were significantly higher for an opposite-sex stranger (potential partner; *M* = 3.57 ± 0.06) compared with a same-sex stranger (*M* = 3.04 ± 0.06). Furthermore, we observed an interaction between target and deafness, *F*(1, 170) = 17.97, *p* < 0.001, *ŋ*^2^ = 0.096, with the follow-up comparisons indicating that significantly greater importance was attributed to the intact modalities in hearing participants compared with deaf participants with regard to an opposite-sex stranger (potential partner; *p* < 0.001). However, this difference was non-significant with regard to a same-sex stranger (*p* = 0.22). In addition, the results revealed a between-target difference in the assessments made by hearing individuals (*p* < 0.001), which was non-significant in deaf participants (*p* = 0.12) (all effects in an omnibus model are described in Table 1). Thus, we conducted two models for each target separately. We considered the effects of deafness, sex, and modality on assessments of two types of targets: an opposite-sex stranger (potential partner) and a same-sex stranger.

**Table 1:** Coefficient of the omnibus repeated-measures full-factorial model examining the effects of target, sex, deafness, and modality on the importance of intact senses in social assessments made by deaf individuals

Factor(s)	*F*	*df* 1	*df* 2	*p*	*η*^2^
*Within-subject effects*					
Target	43.73	1	170	< .001	0.205
Target * sex	.21	1	170	.65	0.001
Target * deafness	17.97	1	170	< .001	0.096
TARGET * sex * deafness	< .001	1	170	.96	< .001
Modality	82.27	2	340	< .001	0.33
Modality * sex	2.63	2	340	.07	0.02
Modality * deafness	22.52	2	340	< .001	0.12
Modality * sex * deafness	2.02	2	340	.13	0.01
Target * modality	.29	2	340	.74	.002
Target * modality * sex	.5	2	340	.75	.002
Target * modality * deafness	1.43	2	340	.24	.008
Target * modality * sex * deafness	.64	2	340	.53	.004
*Between-subject effects*					
Sex	.26	1	170	.61	.002
Deafness	16.26	1	170	< .001	.087
Sex * deafness	.85	1	170	.36	.005

### The Role of Vision, Touch, and Smell in the Assessment of an Opposite-Sex Stranger

**Fig. 1:**
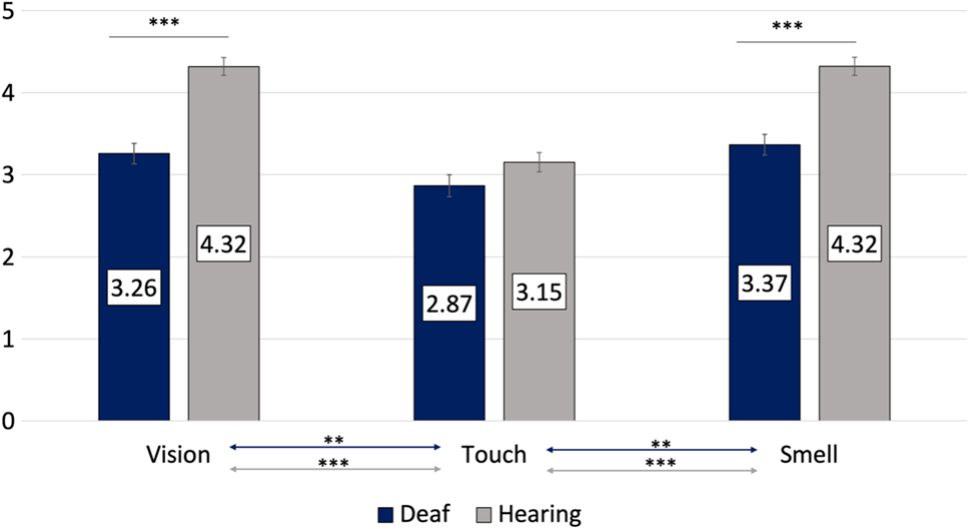
Estimated marginal means for the importance attributed to the intact senses by deaf individuals and their hearing counterparts when assessing an opposite-sex stranger (potential partner). Error bars represent standard mean error. ***p < .001; **p < .01

In the model examining the importance attributed to the intact senses with regard to an opposite-sex stranger (a potential partner) we found a significant interaction effect between modality and deafness, *F*(2, 340) = 9.95, *p* < 0.001, η^2^ = 0.06, suggesting that the overall importance of intact senses was lower in deaf participants compared with their hearing counterparts (*p*s < 0.001) with the exception of touch, which was rated similarly in both groups (*p* = 0.11). Touch had the least importance for attractiveness assessment of an opposite-sex stranger (potential partner). In both groups, vision and smell were rated as similarly important (*p*s > 0.44), and both were more important than touch (all *p*s < 0.006). However, these two modalities were significantly more important to normally hearing individuals, compared with deaf participants (*p* < 0.001). These results are presented in Fig. 1. There were no other significant main or interaction effects.

Deafness had no effect on the three alternative-forced choice of the most important modality in an overall assessment of an opposite-sex stranger (potential partner) *χ*^2^(2) = 0.20, *p* = 0.90 or the most important modality in attractiveness assessment, *χ*^2^(2) = 2.12, *p* = 0.35.

### The Role of Vision, Touch, and Smell in the Assessment of a Same-Sex Stranger

**Fig. 2:**
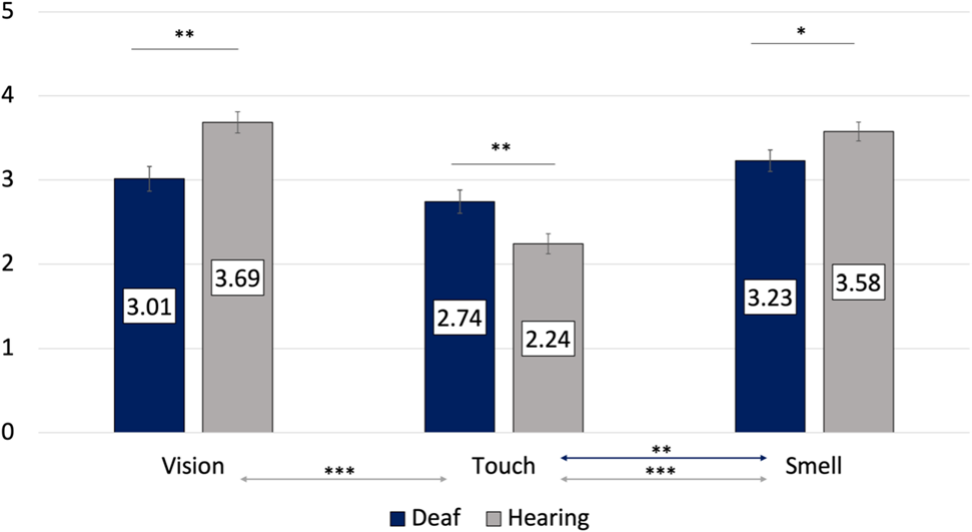
Estimated marginal means for the importance attributed to the intact senses by deaf individuals and their hearing counterparts when assessing a same-sex stranger. Error bars represent standard mean error. ***p < .001; **p < .01

The model examining the effects of deafness and sex on the importance attributed to the intact senses in the assessments of a same-sex stranger yielded a significant interaction effect between modality and deafness, *F*(2, 340) = 18.76, *p* < 0.001, *η*^2^ = 0.10, suggesting that deaf participants attributed significantly lower importance to visual (*p* = 0.001) and olfactory (*p* = 0.042) cues when assessing a same-sex stranger compared with their normally hearing counterparts. However, the reverse was true for tactile cues, which were rated as more important by deaf than normally hearing participants (*p* = 0.006). In deaf participants, touch was rated as significantly less important than olfaction (*p* = 0.001), but not vision (*p* = 0.076), and olfaction was equally important to vision (*p* = 0.171). In contrast, in normally hearing participants, visual and olfactory cues were rated similarly (*p* = 0.428) while touch was rated as significantly less important than both of them (*ps* < 0.001). These effects are shown in Fig. 2.

Deafness had no effect on the three alternative-forced choice of the most important modality in an overall assessment of an same-sex stranger *χ*^2^(2) = 2.92, *p* = 0.23.

## Discussion

In the present study, we examined the role of sensory channels (vision, touch, and smell) in social assessments made by deaf men and women in comparison to normally hearing individuals of a similar age. We hypothesized that the importance of visual and tactile cues would be enhanced as a result of deafness due to previous evidence of enhanced visual and tactile processing in socially relevant tasks among deaf individuals. The current results revealed no support for this hypothesis.

When assessing a stranger in a potential mating situation, both groups rated sensory input as more important, compared with social assessment of a same-sex stranger. Deaf and hearing participants reported the same ranking of modalities: vision was ranked as equally important to smell, and these were both significantly more important than touch. Furthermore, we found that vision and smell were rated as significantly more important by hearing compared with deaf participants, whereas tactile information was found to be equally important for deaf and hearing participants when assessing an opposite-sex stranger (potential partner). For the assessment of a same-sex stranger, deaf individuals reported that touch was more important, compared with hearing controls. These conclusions drawn for the targets separately could potentially signal sensory compensation enhancing the role of touch in social interactions of deaf individuals with same-sex strangers, but the lack of a three-way interaction (deafness × target × modality) in the omnibus analysis of variance model suggests that further studies are required to replicate these results. Interestingly, deaf individuals rated vision and touch as equally important for the assessment of a same-sex stranger, whereas the sense of smell was rated as the most important sense, and was considered significantly more important than touch.

Physical appearance is widely considered to be the most important driver of perceived attractiveness (Stiff et al., 1989). Vision allows a person to read socially relevant traits from another individual’s facial appearance. The face can be a source of information about a person’s trustworthiness, competence or domination (Little et al., 2012; Oosterhof & Todorov, 2008; Todorov et al., 2008, 2009). Even 100 ms of exposure to the human face is sufficient to formulate a judgment about a person (Willis & Todorov, 2006). Intuitive attributions from the human face have a physiological underpinning; positive valence and prosocial traits are more often attributed to feminine and children’s faces, whereas anti-social traits of negative valence are more often attributed to masculine faces (Montepare & Zebrowitz, 1998; Perrett et al., 1998; Zebrowitz & Montepare, 2008). Vision is crucial for assessing silhouette attributes, such as waist-to-hip ratio (WHR), body mass index (BMI) (Fan et al., 2004), and body symmetry, which can all be indicative of attractiveness, general and reproductive health (Dobbelsteyn et al., 2001; Jasieńska et al., 2004; Singh, 1993; Singh & Luis, 1995). However, recent studies indicate that preferences for silhouettes can develop in the absence of visual input (Karremans et al., 2010), suggesting an important role of non-visual input in the perception of attractiveness. In the current study, vision was found to be more important for social assessments by hearing compared with deaf participants, suggesting a valuable interaction between visual and auditory cues that can be fully utilized by hearing individuals when assessing other people in both romantic and social contexts (Groyecka et al., 2017; Zaki, 2013). These results supplement previous findings showing that, when assessing attractiveness, blind individuals shift their attention to auditory cues (Sorokowska et al., 2018). Therefore, vision and audition can compensate for each other. However, blind individuals tend to concentrate on auditory cues, whereas deaf individuals appear to combine visual and olfactory cues to assess attractiveness. Sensory impaired individuals tend to rely on distal senses (vision, audition). Deaf people may support their social judgments with olfactory cues that allow them to draw conclusions about socially relevant characteristics of the target (Lübke et al., 2014; Mahmut et al., 2019; Porter et al., 1986; Roberts et al., 2011; Sorokowska, 2013a; Sorokowska & Oleszkiewicz, 2019; Sorokowska et al., 2016) while keeping at least a minimal interpersonal distance. In contrast, touch requires direct physical contact with the target. Another plausible explanation is related to the reliability of the type of sensory cues in social cognition. Audition is preferred by blind individuals in social judgment, and olfaction, additionally engaged by deaf people, may be more difficult to intentionally manipulate in self-presentation by a target than visual appearance. Therefore, auditory and olfactory cues may be preferred by blind and deaf individuals to build a more accurate perception of the target in a social context.

The current results highlight the importance of the sense of smell in social cognition processes, which has received relatively little research attention (but see: Bendas et al., 2018; Drea, 2015; Lübke & Pause, 2015; Pause, 2011). Olfactory cues play an important role in regulating human relationships. The human sense of smell drives overall attractiveness judgments (Thornhill et al., 2003) and provides signals of genetic incompatibility of partners (Wedekind & Füri, 1997; Wedekind et al., 1995), which further highlights the importance of chemosignalling in human mate choice. Beyond mating, the human nose uses body odor as a premise for social judgments, particularly in the recognition of individuals in terms of personal identity (Penn et al., 2006), fear (Albrecht et al., 2010; Chen, 2006; de Groot et al., 2012; Prehn et al., 2006; Zernecke et al., 2011; Zhou & Chen, 2009), happiness (Chen & Haviland-Jones, 2000; de Groot et al., 2015), and reactions to disgust (de Groot et al., 2012). Furthermore, people can use olfactory cues to relatively accurately attribute biological qualities such as sex (Hold & Schleidt, 2010; Schleidt, 1980), and psychological traits such as neuroticism and dominance (Sorokowska et al., 2012; Sorokowska, 2013a, b). Moreover, dominance (Havlicek et al., 2005; Rantala et al., 2006; Sorokowska, 2013a) and neuroticism (Sorokowska, 2013a) additionally affect the perception of body odor attractiveness. Olfactory stimulation also plays a role in shaping romantic relationships. Loss of the sense of smell may result in social insecurity. Men with anosmia reported fewer sexual relationships in their life, whereas women with anosmia were reported to feel less secure about their partners (Croy et al., 2013). The results of the current study further corroborate this line of investigation by demonstrating that, besides vision, the sense of smell is reported to be important by individuals without auditory input, as well as their fully functional counterparts in both mating and non-mating social contexts.

The current results revealed that touch was the least important input for forming impressions of others. Different forms of touch convey different meanings (Burgoon, 1991), and may be viewed differently depending on gender (Nguyen et al., 1975). For example, a handshake, the most common type of touch exchanged between two strangers, expresses formality and trust (Burgoon, 1991). However, there are other forms of touch, such as handholding, face touching, and hugging, which express emotional attachment. Touch transmits sensations and emotions (Morrison et al., 2009) causing both physiological and psychological reactions (McGlone et al., 2014) described as crucial for child development and avoiding somatosensory deprivation (Prescott, 1975). Interpersonal touch enhances social bonding (Dunbar, 2010) through activation of the endogenous opioid system in humans (Nummenmaa et al., 2016). Touch is an intimate contact that requires infringing on interpersonal distance, and, as a consequence, creates a situation of vulnerability (Thayer, 1986) that can transform into mutual trust (Colquitt et al., 2007). In many situations, touching someone requires prior knowledge of the person and an established relationship. For this reason, our participants might have found it less important for building impressions of others compared with vision and olfaction. However, in the case of a same-sex stranger, deaf participants declared that greater attention was paid to tactile stimulation than normally hearing controls. One potential reason for this is the process of learning and using sign language by deaf participants, who might touch others and get touched by them in this situation. Touching others might be more natural, and is potentially helpful for expressing thought or needs when deaf people talk with each other, or when a deaf person talks to a hearing partner but is not able to vocalize properly. The role of sign language in the perception of interpersonal touch warrants further study.

One potential limitation of the current study is related to the nature of the procedures we applied, basing our conclusions on self-reported data. Although this method is widely used in psychological research, we gathered our data at a single time-point. A follow-up session would be required to assess the reliability of the results. Some previous studies have suggested that olfactory cues are overestimated when self-reported, and that vision plays a stronger role when confronted with actual stimuli (Foster, 2008). To verify the current findings, it may be helpful for future studies to examine this issue in real-life social and mating interactions and capture real and not only declared preferences. However, this may be difficult in the deaf community, in which such experiences may be particularly private, and individuals may not be willing to expose delicate social interactions (e.g., mating) to researchers. Future studies on the importance of the senses in social and mating contexts could benefit from engaging neuroscientific techniques to gain more objective insights that could supplement self-report data. Another potential limitation was the presence of sign language translators, whose presence was critical for reliable communication with our participants, but could have resulted in social desirability bias. We did not match translator-participant sex in any way. To provide a comfortable and friendly atmosphere during the meeting, sign language translators were personally well acquainted with the deaf participants. Although we did not control for the sexual orientation of the participants, we believe there is no reason to suppose it had any substantial influence on the importance attached to the particular modalities. Finally, the presented research question should be explored in the context of the length of a potential relationship (short- vs long-term) to examine whether visual, olfactory, and tactile premises are similarly valued in relationships of different lengths.

### Conclusion

According to the World Health Organization (2019), approximately 5% of the population suffers from hearing loss. Little is known about how deaf people construct their social relationships and how they build impressions of others. In the current study, we investigated this issue from a mating and social perspective. The relationship between deafness and the sensory basis of social judgment did not reveal a simple increase in the level of attention paid to all stimuli other than audition. Rather, our findings suggested that deaf individuals rated input from the intact modalities as less important for social assessments than normally hearing controls, yet the relative importance of vision, olfaction, and touch remained similar in both groups. Although vision and olfaction were most important in perception of others, touch remained significantly more important in assessments of a same-sex stranger, potentially indicating an increased role of tactile communication in signers.
